# The Fatal Ambition

**Published:** 1871-10

**Authors:** 


### THE FATAL AMBITION.

She aimed to graduate at the head of the class, and succeeded. For weeks and months she gave her whole time, and thoughts, and energies to the one great object of preparing herself to pass a creditable examination, a fear hanging over her all the while that she might "fail" and be "rejected." She gave herself no time for recreation; she curtailed the hours of sleep, ate rapidly, and studied intensely. On the evening of the day of her examination she was taken ill; the illness increased; in ten days she was buried. Such is the history of the accomplished daughter of one of our best citizens.

A similar history may be written of many a theological student, and of young gentlemen designing to enter other professions and callings.

Similar fatalities befall those who overstrain the muscular system for purposes of competition, as in the case of Renforth the boatman. Competitive study, and competitive games, are equally pernicious and are very dangerous; for either fatal reaction takes place, and the system falls into a typhoid condition, or the strain reaches the utmost of its endurance, and the cord of life snaps asunder.

None, especially the young, should be hurried in mental or bodily exercise; they should work by the day instead of by the job. Most persons working by themselves, especially if educated, are almost sure, when they have anything to do, to attempt to do it in the shortest time possible; it is a great and prevalent and often a fatal error. Only deliberate labor is healthful. Hurry is pernicious to mind and body. Just as injurious is long continued study and long continued work. The man or woman who works or studies to the point of almost utter exhaustion is a virtual suicide.

